# The Impact of Empowering Leadership on Taking Charge Behaviors: Mediating Strengths Use and Moderating Ambition

**DOI:** 10.3390/bs14080633

**Published:** 2024-07-24

**Authors:** Lingnan Kong, Yang Liu, He Ding, Sha Liu, Shunkun Yu

**Affiliations:** 1 School of Economics and Management, North China Electric Power University, No. 2, Bei Nong Road, Changping District, Beijing 102206, China; 2 National Engineering Research Center of New Energy Power Generation, North China Electric Power University, No. 2, Bei Nong Road, Changping District, Beijing 102206, China

**Keywords:** empowering leadership, ambition, strengths use, taking charge behavior

## Abstract

Drawing on conservation of resources theory, this study aims to explore the mediating role of employees’ strengths use and the moderating role of employees’ ambitions in the relationship between empowering leadership and employees’ taking charge behaviors. A total of 333 Chinese employees from various organizations across China (including industries such as manufacturing, IT, and education) completed our survey at two time points, with a two-week lag. We used structural equation modeling and moderated mediation path analysis to test our hypotheses. The research revealed that strengths use partially mediated the relationship between empowering leadership and employees’ taking charge behaviors, and ambition positively moderated the relationship between empowering leadership and strengths use, as well as the indirect relationship between empowering leadership and employees’ taking charge behaviors through strengths use. It extends the application field of strengths use, providing a new theoretical perspective on behavioral mechanisms for understanding the empowering leadership–employees’ taking charge behaviors relationship, and offers valuable strategies for organizations and leaders to promote employees’ taking charge behaviors more effectively.

## 1. Introduction

In the context of the rapid development of the globalized economy, enterprises are in a highly dynamic, complex, and competitive environment, facing the challenges of rapid technological iteration and transformation [1,2,3,4,5]. To cope with these rapid changes in the business environment and achieve sustainable development, companies expect employees to engage in taking charge behavior, promoting functional changes within the organization to enhance the company’s ability to face environmental challenges [6,7,8]. Taking charge is an extra-role behavior that employees voluntarily engage in to facilitate functional organizational changes [9,10,11], occurring in their jobs, work units, and organizations [9,12]. It not only helps improve organizational performance but also stimulates organizational creativity, and enhances organizational vitality and adaptability [6,7], which are crucial for maintaining a sustained competitive advantage. However, because it is challenging and risky, it requires voluntary and creative efforts from employees, which leads many employees to not undertake it on their own initiative. Investigating how to stimulate employees’ taking charge behaviors to promote constructive changes in the work methods of individuals, groups, or organizations is of great significance for the high-quality sustainable development of enterprises [11,13,14,15,16,17]. Especially considering the context of China, the country is in an economic transition period [18,19,20], and enterprise development requires employees to invest more in taking charge behaviors. Moreover, for employees, engaging in taking charge behaviors not only helps achieve higher performance but also enhances their job satisfaction [8,13,14], benefiting their long-term career development [11]. Therefore, encouraging taking charge behaviors among employees holds significant importance for both the organization and its employees.

Leadership, as a critical factor influencing employee behavior, has attracted the attention of many scholars. For example, existing research has confirmed that transformational leadership [2,16,21], inclusive leadership [22], and authentic leadership [23] positively impact employees’ inclination toward taking charge. Empowering leadership [24,25,26,27], as a positive leadership style, can enhance the sense of work value, encourage employee participation in decision-making, express confidence in high employee performance, and provide autonomy beyond hierarchical levels, positively affecting employee creativity [28,29], knowledge sharing [30], and advisory behavior [31]. Consequently, the relationship between empowering leadership and taking charge has also sparked scholarly interest. Existing research has found that empowering leadership can affect taking charge by influencing employees’ psychological empowerment [10,32] or by affecting employees’ thriving at work, which, in turn, impacts employees’ change-oriented organizational citizenship behavior [33]. However, scholars have often explored this from a motivation perspective [10] or a combined emotion–cognition perspective [10,33], yet research on the behavioral influence mechanism between empowering leadership and employees taking charge remains unexplored. Therefore, the primary goal of this study is to investigate, from the perspective of conservation of resources (COR) theory, whether empowering leadership can influence employees’ taking charge behaviors through behavioral mechanisms, which is of great research value.

Strengths use, as an important individual resource, refers to the behavior of employees proactively using their strengths to complete work tasks [34], which can function similarly to work resources. According to COR theory, individuals strive to protect their existing resources and acquire new ones [35]. Empowering leadership can act as a job resource, with strengths use being an individual behavioral resource [36]. Based on COR theory, it can be reasonably inferred that strengths use may mediate between empowering leadership and employees’ taking charge behaviors. For one thing, strengths use, as an action taken by employees to leverage their advantages at work [34], has been shown in numerous empirical studies to lead to various desirable outcomes, such as increased work engagement [37,38], work performance [39,40], task performance [41], innovative behaviors [42], and organizational citizenship behaviors [43]. Furthermore, strengths use is an important motivator for human well-being [44], which is crucial for sustainable development [45]. Yet, the literature is lacking in regard to examining the relationship between strengths use, empowering leadership, and taking charge behavior. For another thing, there is an intrinsic connection between the essence of empowering leadership and strengths use. Leaders may not always be aware of the individual strengths of their employees; when leaders grant autonomy, it creates space for employees to utilize their strengths and increases their motivation to apply these strengths to solve work-related problems. Therefore, exploring how the individual resource of strengths use can serve as a mediator between empowering leadership and employees’ taking charge behaviors helps to fill this gap in the literature. Our second research goal is to test whether employees’ strengths use mediates the impact of empowering leadership on employees’ taking charge behaviors.

The third research goal is to explore the moderating role of employees’ ambition between empowering leadership and employees’ use of strengths. Employee ambition [46] is an individual’s drive for achievement and recognition, which can have a broad impact on career behavior [47,48]. We posit that employee ambition may moderate the relationship between empowering leadership and employee strengths use. This is because, according to COR theory, Halbesleben et al. [49] define the value of a resource as the extent to which an individual’s resource can promote the achievement of his goals. Employees with high ambition are more likely to view the empowering actions of leadership as valuable resources, including recognition and career advancement. They may leverage their strengths to achieve positive outcomes. Based on this theoretical rationale, we hypothesize that employees with higher levels of ambition are more inclined to fully utilize their strengths in taking proactive change actions. Given that employees’ ambition might modulate the impact of empowering leadership on the use of strengths, it is plausible to believe that employees’ ambition could enhance the indirect effect of empowering leadership on employees’ taking charge through strengths use.

To summarize, we propose three research goals in this paper, aiming to explore why, how, and when empowering leadership affects employees’ taking charge behavior based on COR theory, introducing employees’ strengths use as a mediator and ambition as a moderator, filling current literature gaps and making three theoretical contributions. Initially, from the perspective of COR theory, this paper explores why empowering leadership can influence employees’ taking charge behavior, further supplementing the literature on the relationship between empowering leadership and taking charge behavior. Furthermore, by introducing strengths use as a mediating variable, this paper deepens our understanding of the potential behavioral mechanisms underlying the relationship between empowering leadership and taking charge behavior. Finally, by examining the positive moderating influence of employee ambition, this paper enables a better understanding of when empowering leadership is more effective in promoting employees’ taking charge behavior through strengths use.

## 2. Theory and Hypotheses

### 2.1. Empowering Leadership and Taking Charge

Taking charge, as a challenging and risky extra-role behavior that employees voluntarily engage in to promote organizational functional change [9], shares a voluntary nature with organizational citizenship behavior but focuses more on change orientation and constructiveness [7,50,51]. Research has shown that personal factors of employees (such as self-efficacy) [9], leadership factors (such as leaders’ outcome control over subordinates) [52], and situational factors (such as procedural justice [6] and leader–member exchange [13]) all influence employees’ taking charge. Therefore, focusing on the impact of leadership style on employees’ taking charge is of significant research value.

Empowering leadership is defined as actions aimed at enhancing employee performance by granting subordinates authority and focusing on empowerment practices within the organization [26]. These practices include delegating power, emphasizing responsibility, and fostering autonomy in decision-making [24,25,26,27]. COR theory posits that when employees acquire resources from their work, a balance in resource exchange between employees and the organization needs to be achieved, with employees striving to reach this balance [35,53]. By enhancing the importance of work, facilitating participation in decision-making, expressing confidence in employees’ high performance, and providing autonomy [24], leaders offer the work and psychological resources needed for taking charge [6,9,54], essential for employees facing the inherent uncertainties and challenges of initiating change [55,56,57]. For one thing, the array of empowering behaviors from leaders, providing necessary work resources for employees to refine work methods and organizational processes among other change-oriented actions, enables employees to adjust their strategies and methods more flexibly. For another thing, empowering leadership offers psychological resources to employees, bolstering their confidence to tackle risks associated with change. Based on COR theory, a series of empowering actions by leaders can be seen as valuable resources that employees can utilize. When employees feel that resources are abundant, they may engage in more taking charge behaviors, thereby achieving a balance in resource exchange between employees and the organization. Furthermore, engaging in taking charge behaviors depletes the valuable resources of employees, and those who are more empowered may exhibit higher levels of taking charge, mainly because the authorization from leaders provides critical resources for employees to carry out such changes. Therefore, based on the above argument, we propose the following hypothesis:

**Hypothesis** **1.** *Empowering leadership is positively related to employees’ taking charge behavior.*

### 2.2. The Mediating Effect of Strengths Use

Strengths use is an individual resource that is as important as work resources for employees [34]. Employees become happier, more confident, and more creative [39], and they experience higher job satisfaction [58] when they engage in work that allows them to use their strengths. Research indicates that using strengths positively impacts employee innovation [42], work engagement [37,38], work performance [39,40], task performance [41], and organizational citizenship behavior [43]. Therefore, it is very valuable to explore whether strengths use has an impact on employees’ taking charge.

According to COR theory, work resources contribute to the increase in personal resources [59]. Furthermore, the theory highlights the presence of a “gain spiral”, meaning that when individuals have ample resources, they have greater chances to gain new resources through investing their existing ones, enhancing their resource pool, and nurturing more subsequent resource growth, forming a spiral [60]. For one thing, empowering leadership, as a positive leadership style, makes employees feel that their contributions are valued and recognized. This recognition boosts their self-efficacy and confidence in their abilities, encouraging them to use their strengths more frequently and effectively within their roles [28]. When leaders express confidence in employees’ abilities, it acts as a form of psychological empowerment, motivating employees to undertake tasks that align with their strengths [61]. For another thing, the autonomy granted by leader empowerment allows employees to adjust their work roles based on their strengths. By giving employees the freedom to decide how to complete their work, leaders enable them to design their work in a way that maximizes their personal and professional strengths. Empowering leadership not only provides the necessary resources for employees but also establishes a cycle of resource gain through empowerment, further facilitating the accumulation of personal resources by employees. Based on the above reasoning, it is reasonable to hypothesize that empowering leadership has a positive effect on employees’ use of their strengths.

Moreover, COR theory suggests that when employees gain resources from their work, a balance in resource exchange between the employees and the organization is necessary, with employees endeavoring to reach this equilibrium [35,53]. Since the use of strengths by employees can be considered an important work resource [62], employees who utilize their strengths in work may engage in more taking charge behaviors to achieve a balance in resource exchange between themselves and the organization. Therefore, employees with a higher level of strengths use may exhibit a higher level of taking charge. Furthermore, research has found that employees’ use of strengths provides important resources for organizational citizenship behavior, and studies have also found a positive correlation between strengths use and organizational citizenship behavior [43]. Given that both organizational citizenship behavior and taking charge are extra-role behaviors, it can be inferred that strengths use may also have a positive relationship with taking charge. Considering that strengths use can be regarded as a personal resource [63], there is reason to believe that employees using their strengths will help provide more of the individual resources needed for taking charge.

In summary, leaders provide employees with work and psychological resources, and employees utilize their strengths, creating a positive spiral of resource growth. This makes employees more willing to engage in resource exchange with the organization to achieve balance, such as by taking charge, which depletes their resources. Based on the above reasoning, we propose the following hypothesis:

**Hypothesis** **2.** *Strengths use mediates the relationship between empowering leadership and employees’ taking charge behavior.*

### 2.3. The Moderating Effect of Ambition

Employee ambition is defined as a persistent and generalized striving for success, attainment, and accomplishment [64]. It is seen as a relatively stable personal disposition [48]. The key characteristic of ambition includes a drive for success. Ambition is associated with setting challenging goals, striving for outstanding results, and accomplishing great tasks [64]. It is not just about climbing the career ladder but encompasses a broader desire to achieve and be recognized for one’s achievements [65,66,67]. Previous studies have indicated that employee ambition has a significant effect on job performance and organizational commitment [64,68,69]. However, there is currently a lack of research on the relationship between employee ambition and leadership style, so it is meaningful to investigate employee ambition as a moderating variable in this study.

Based on COR theory, individuals endeavor to preserve, protect, and amass resources they find valuable [49]. Halbesleben et al. defined the value of a resource as an individual’s estimate of the extent to which a resource can facilitate the achievement of their goals, further noting that an individual’s valuation of a resource determines whether and how they preserve and acquire that resource [49]. Employees with high ambition, due to their high pursuit of challenging goals and success, are more likely to consider the empowering actions of leaders as valuable resources for personal career advancement and achievement, thereby utilizing their strengths. Ambitious employees achieve a spiraling growth of resources by accessing resources empowered by leaders and utilizing their strengths. Based on these, we speculate that employee ambition reinforces the positive relationship between empowering leadership and the utilization of employee strengths.

Therefore, the following hypothesis was obtained:

**Hypothesis** **3.** *Ambition enhances the relationship between empowering leadership and strengths use in such a way that the relationship is more positive for employees with high ambition than for employees with low ambition.*

Up to now, we have proposed Hypotheses 1–3. Given inherently ambitious employees’ quest for high levels of success and recognition, their willingness to embrace empowering leadership, and proactive utilization of their strengths, they are likely to more eagerly and voluntarily innovate work methods and workflows and embrace the challenges brought about by change, viewing them as avenues to personal career success and accomplishments. Based on these hypotheses, we further postulated that the mediational effect of strengths use on the relationship between empowering leadership and employees’ taking charge behavior is contingent on employees’ ambition. Thus, we postulated:

**Hypothesis** **4.** *Ambition enhances the indirect relationship between empowering leadership and taking charge via strengths use such that the indirect relationship is more positive when ambition is high than low.*

**Figure 1:**
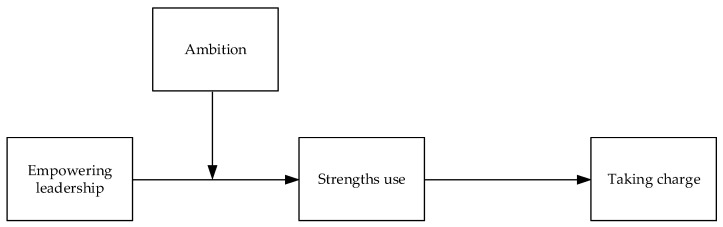
The proposed research model.

The proposed conceptual model is depicted in Figure 1.

## 3. Method

### 3.1. Data Collection and Study Sample

This study employed a convenience sampling strategy to enlist employees from diverse industries, such as manufacturing, information technology, and education, across various organizations in China. Data collection was conducted through a Chinese online questionnaire platform named Credamo. Before data collection, participants were assured of strict confidentiality regarding their information. They were informed of their right to withdraw from the survey at any point and were granted unrestricted access to the study’s findings. Since the questionnaires were all self-reported by employees, to prevent common method variance, the survey was conducted in two phases with a two-week interval. According to Podsakoff et al.’s recommendation [70], this method of collecting data at two phases with a two-week interval is a procedural control measure and one of the effective methods to avoid common method variance. This method has also been applied in other empirical studies [58,71].

The first-phase questionnaire was directly sent to the subjects who had registered on the survey platform with a unique identification number. At Phase 1, 500 questionnaires were distributed and 412 were collected, with a response rate of 82.40%, establishing a sample database. In this phase, the participants were instructed to join a questionnaire survey regarding demographic variables (e.g., age, gender, education, tenure, and job level), and including an empowering leadership scale and an ambition scale. Two weeks later, using the “Targeted Tracking Survey” feature provided by the survey platform, a second-phase questionnaire was directed to the subjects in the sample database, with 355 collected, resulting in a response rate of 86.17%. These participants at Phase 2 were asked to complete questionnaires concerning the strengths use scale and the taking charge scale. Additionally, considering that senior leaders may not have direct supervisors, we excluded 22 senior leaders from the samples collected in the second-phase, resulting in a final sample of 333, including ordinary employees, line leaders, and middle leaders. The sample characteristics distribution is displayed in Table 1.

### 3.2. Measurement Scales

To adapt the constructs of empowering leadership, ambition, strengths use, and taking charge for Chinese participants, which were originally developed in English, we employed the translation and back-translation method proposed by Brislin [72]. This approach ensured the accuracy and cultural relevance of the Chinese versions of these scales. Responses for all scale items were captured using a 5-point Likert scale, with options ranging from 1 (strongly disagree) to 5 (strongly agree).

***Empowering leadership*.** We measured empowering leadership with a 12-item scale developed by Ahearne et al. [24]. An example item was “My manager allows me to do my job my way”. The questionnaire item scale is displayed in Appendix A. Cronbach’s α was 0.89.

***Ambition.*** Ambition was evaluated with a 5-item scale developed by Hirschi and Spurk [46]. An example item was “For me, it is very important to achieve outstanding results in my life”. The questionnaire item scale is displayed in Appendix A. Cronbach’s α was 0.88.

***Strengths use.*** Employee strengths use was evaluated with 5 items used by Ding and Lin [71]. An example item was “In my job, I try to apply my talents as much as possible”. The questionnaire item scale is displayed in Appendix A. Cronbach’s α was 0.76.

***Taking charge*.** Based on the 10 measurement items compiled by Morrison and Phelps [9], this paper draws on Li et al.’s [73] measurement methods of taking charge in the Chinese context, and selects 6 items with the highest factor load coefficient from the original scale to measure taking charge. An example item was “I often try to correct a faulty procedure or practice”. The questionnaire item scale is displayed in Appendix A. Cronbach’s α was 0.90.

***Control variables***. According to previous literature [10,23], the current study controlled for gender (0 = male, 1 = female), age (years), organizational tenure (years), education (0 = under bachelor’s degree, 1 = bachelor’s degree, 2 = master’s degree, 3 = doctor’s degree), and job level (0 = ordinary employee, 1 = line manager, 2 = middle manager).

## 4. Data Analyses and Results

### 4.1. Confirmatory Factor Analysis

Before testing our hypotheses, consistent with previous studies [43,74], confirmatory factor analysis was conducted in AMOS 24 to examine the discriminant validity of empowering leadership, ambition, strengths use, and taking charge. Table 2 presents the fit indices for various measurement models. The analytical findings indicated that the four-factor measurement model demonstrates a superior fit to the data compared to alternative models, thereby confirming the discriminant validity of the four central research variables.

Despite data collection occurring at two separate time points, the potential for common method variance necessitates further examination due to the reliance on a single data source. Following Podsakoff et al.’s recommendation [70], a single unmeasured latent method factor approach was employed to assess the presence of common method variance in the research data. A method factor was created and then loaded on all items of empowering leadership, ambition, strengths use, and taking charge. A five-factor measurement model, which included the method factor along with the four principal constructs of this study, demonstrated a superior fit to the data ($\chi^{2}$ = 677.88, df = 343, $\chi^{2}/df$ = 1.98, CFI = 0.93, TLI = 0.92, IFI = 0.93, RMSEA = 0.05) compared to a four-factor model. However, the method factor accounted for only 12.74% of the variance, which is below the 25% threshold suggested by Williams et al. [75]. Consequently, common method variance does not pose a significant threat to the validity of our findings.

### 4.2. Descriptive Statistics and Correlational Analysis

Table 3 reports the means, standard deviations, and correlation coefficients among gender, age, education, tenure, job level, empowering leadership, ambition, strengths use, and taking charge. The results of the correlational analysis showed that empowering leadership is positively related to ambition (r = 0.62, *p* < 0.01), strengths use (r = 0.62, *p* < 0.01), and taking charge (r = 0.67, *p* < 0.01); ambition is positively related to strengths use (r = 0.54, *p* < 0.01), and taking charge (r = 0.60, *p* < 0.01); strengths use is positively related to taking charge (r = 0.68, *p* < 0.01). These results offered initial support for our hypotheses.

### 4.3. Hypotheses Testing

In order to examine the hypotheses, similar to previous research methods [76,77], we used structural equation modeling (SEM) to validate and analyze our model. We averaged the items of each dimension of empowering leadership and treated the different dimensions as separate indicators of this focal variable in the SEM analysis. For the other three focal variables—ambition, strengths use, and taking charge—we used their individual items as observed variables for measuring the latent variables.

Hypothesis 1 posited a positive relationship between empowering leadership and taking charge behaviors. To test this hypothesis, we implemented SEM analysis in AMOS 24, complemented by a bootstrapping technique with 10,000 resamples, and used 95% bias-corrected confidence intervals to assess the significance of direct pathways. We established a structural equation model (Model 1) in which age, gender, education, tenure, job level, and empowering leadership, respectively, predict taking charge. Analytical results indicated that this model fits the data very well ($\chi^{2}$ = 159.21, df = 84, $\chi^{2}/df$ = 1.90, CFI = 0.97, TLI = 0.97, IFI = 0.97, RMSEA = 0.05, SRMR = 0.06). The path coefficient between empowering leadership and taking charge was significant (estimate = 0.63, 95% CI: [0.49, 0.77], *p* < 0.001). Therefore, Hypothesis 1 received support.

**Figure 2:**
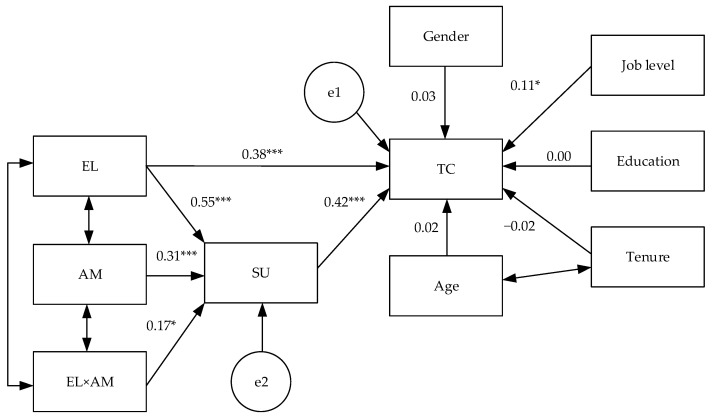
The results of the moderated mediation path analysis. Note: EL = Empowering leadership, AM = Ambition, EL × AM = Interaction of empowering leadership and ambition, SU = Strengths use, TC = Taking charge; *** *p* < 0.001, * *p* < 0.05.

To examine Hypotheses 2–4, we established a moderated mediation model (Model 2), in which strengths use as a mediator and ambition as a moderator were introduced. The interaction term of empowering leadership and ambition was treated as an explicit variable. Control variables, concerning gender, age, education, tenure, and job level, were also used to predict taking charge. We further carried out a bootstrapping analysis, resampling 10,000 times, and utilized 95% bias-corrected confidence intervals to evaluate the significance of both direct and indirect paths. Analytical results demonstrated that this moderated mediation model fits the data very well ($\chi^{2}$ = 602.04, df = 208, $\chi^{2}/df$ = 2.15, CFI = 0.94, TLI = 0.92, IFI = 0.94, RMSEA = 0.06, SRMR = 0.06). The direct path coefficients are displayed in Figure 2. The indirect relationship of empowering leadership with taking charge via strengths use was significant (estimate = 0.23, 95% CI: [0.15, 0.33], *p* < 0.001), supporting Hypothesis 2. Because the direct relationship between empowering leadership and taking charge was still significant (estimate = 0.37, 95% CI: [0.22, 0.52], *p* < 0.001) after introducing strengths use as a mediator, strengths use partially mediates the relationship of empowering leadership with taking charge.

**Figure 3:**
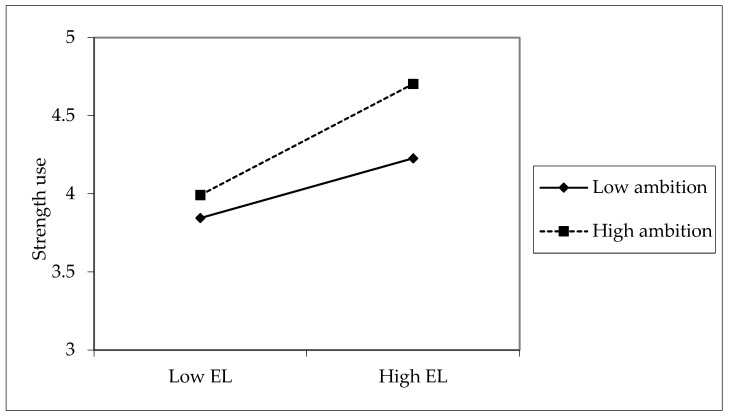
The interaction plot of empowering leadership and ambition on strengths use. Note: EL = Empowering leadership.

Hypothesis 3 postulated that ambition enhances the relationship between empowering leadership and strengths use. The interaction term was significant (estimate = 0.17, 95% CI: [0.03, 0.26], *p* < 0.05). To further explore the interaction effect, we illustrated the relationship between empowering leadership and ambition on strengths use according to Figure 3. Additionally, we performed a simple slope analysis to examine this interaction. The results showed that the positive relationship between empowering leadership and strengths use was significant when ambition was high (M + SD, estimate = 0.71, 95% CI: [0.51, 0.91], *p* < 0.001). When ambition was low, the positive relationship between empowering leadership and strengths use was also significant (M − SD, estimate = 0.38, 95% CI: [0.23, 0.54], *p* < 0.001). And this difference was significant (M − SD vs. M + SD, difference estimate = −0.33, 95% CI: [−0.51, −0.06], *p* < 0.05). In total, Hypothesis 3 received support.

Hypothesis 4 postulated that ambition strengthens the indirect relationship of empowering leadership with taking charge through strengths use. The moderated mediation effect was significant (estimate = 0.07, 95% CI: [0.01, 0.13], *p* < 0.05). Further, the indirect effect of ambition on taking charge via strengths use was 0.16 (M − SD, 95% CI: [0.10, 0.24], *p* < 0.001) at a low level of ambition; the indirect effect of ambition on taking charge via strengths use was 0.30 (M + SD, 95% CI: [0.18, 0.45], *p* < 0.001) at a high level of ambition. And this difference was significant (M − SD vs. M + SD, difference estimate = −0.14, 95% CI: [−0.25, −0.03], *p* < 0.05). Therefore, Hypothesis 4 received support.

## 5. Discussion

Drawing on COR theory, this study of 333 employees examined the impact of empowering leadership on employees’ taking charge behavior and the mediating role of employee strengths use as well as the moderating role of ambition in the relationship. All hypotheses received support from the research data.

### 5.1. Theoretical Implications

This study enriches the theory and research on empowering leadership and taking charge in two significant ways.

First, this article provides a new theoretical perspective on the positive impact of empowering leadership on taking charge, grounded in the COR theory framework, enriching the literature on the relationship between empowering leadership and taking charge. A meta-analysis by Lee et al. indicated that the main effects of empowering leadership on work outcomes (e.g., task performance, organizational citizenship behavior, and creativity) are predominantly positive [78]. Scholars have found that empowering leadership enhances various desirable work outcomes by promoting psychological empowerment [28,61,79], indicating that psychological empowerment often plays a crucial mediating role. Furthermore, in previous studies, Li et al., drawing from the theory of the socially embedded model, discovered that empowering leadership could impact employees’ work prosperity, subsequently influencing their change-oriented organizational citizenship behavior [33], while Qian et al., drawing on social exchange theory, identified that seeking feedback mediates the relationship between empowering leadership and proactive actions, like taking charge [80]. However, it remains unclear how empowering leadership influences taking charge through behavioral mechanisms. This paper, under the COR theoretical framework, explores how empowering leadership influences employees’ taking charge behaviors through strengths use. By examining the mediating role of strengths use, the study explores the behavioral influence mechanism between empowering leadership and employees’ taking charge, filling a gap in the current literature and contributing to a better understanding of how empowering leadership positively affects employees’ taking charge.

According to COR theory, work resources contribute to increase in personal resources [59,60]. And there is a gain spiral, which means that when individuals have abundant resources, they have more opportunities to acquire new resources through resource investment, increasing their resource reserves, and fostering more subsequent resource growth [60]. This study found that empowering leadership not only expresses confidence in employees and provides psychological resources but also grants job autonomy and provides work resources [24,61], all of which further promote the accumulation of personal resources in employees, helping them establish a gain cycle.

Additionally, COR theory posits that when employees obtain resources from their work, there needs to be a balance in the resource exchange between employees and the organization, and employees will endeavor to achieve this balance [35,53]. Given strengths use can be considered an important work resource [62], employees who leverage their strengths at work may undertake more taking charge behaviors to balance the resource exchange between themselves and the organization. Existing research has demonstrated that strengths use promotes organizational citizenship behavior [43], which is also considered as a kind of extra-role behavior. Our study demonstrates that after empowering leadership provides resources to employees, they proactively use their strengths to engage in taking charge behaviors. Therefore, this paper reveals the mediating effect of strengths use, uncovering the “black box” of the behavioral influence mechanism between empowering leadership and employees’ taking charge.

Second, this study examines the moderating role of employee ambition in the relationship between empowering leadership, strengths use, and taking charge, finding that empowering leadership is more effective in enhancing employee initiative in taking charge through strengths use when employees have high ambition. Empowering leadership has been proven to effectively promote positive behaviors in employees, but it does not benefit all employees equally. According to COR theory, individuals strive to maintain, protect, and accumulate resources that are valuable to them [49]. Halbesleben et al. defined resource value as an individual’s estimation of how much a particular resource can promote their goal achievement and pointed out that an individual’s judgment of the value of a resource determines whether and how they conserve and acquire that resource [49]. Ambition is seen as a relatively stable personal disposition [48]. Highly ambitious employees, in pursuit of success, are more willing to set challenging goals and strive for excellent results [64,65,68]. Employees with high ambition achieve a spiraling increase in resources by accessing resources provided through leadership empowerment and utilizing their strengths, which, in turn, prompts them to engage in proactive behaviors, such as taking charge. This discovery aids in a better understanding of the boundary conditions under which empowering leadership more effectively promotes taking charge through employee strengths use in the presence of employee ambition.

### 5.2. Managerial Suggestions

This study has several practical implications. Firstly, taking charge behaviors can significantly enhance job performance [13,81,82], and positively affect employee work engagement [83], job satisfaction [14], and affective organizational commitment [14]. Additionally, it is beneficial to the career development of employees. Given the positive influence of leadership style on taking charge, organizations can foster employees’ initiative in taking charge by selecting, promoting, or developing leaders skilled in empowering. Secondly, by designing and implementing management policies and practices that facilitate the use of strengths [36], employees can be encouraged to maximize their strengths in an autonomous environment. Third, fully leveraging the role of leader empowerment, especially for ambitious employees, can encourage them to use their strengths resources to engage in a series of positive behaviors beneficial to personal career development and organizational progress. By doing so, the effectiveness of leader empowerment may be better realized.

Through a series of initiatives involving leadership empowerment and strengths use by employees, organizations stimulate employees’ innovation and change capabilities, thus facilitating the achievement of sustainable development goals. Moreover, organizations encourage employees to develop professional skills based on their own strengths, which not only aids in their career growth but also accelerates the achievement of organizational goals, particularly in terms of sustainability.

### 5.3. Limitations and Directions for Future Research

This article is not without limitations. Firstly, the data source of this study is singular, which might lead to common method bias. Although we ascertain that the common method bias of this study does not pose a serious threat to our results, future research should attempt paired studies, letting managers assess employees’ strengths use and taking charge. Secondly, we only examined the effects of empowering leadership on employees’ initiative in taking charge at the individual level. Given that teams have become the main working units in current organizations, it is necessary to examine whether team-level empowering leadership also has predictive value for employees’ taking charge in future research. Thirdly, extensive research has found that employees’ initiative is influenced by different leadership styles, such as transformational leadership [2,21], inclusive leadership [22], and authentic leadership [23], which were not controlled for in our study. Therefore, future research should examine whether empowering leadership has additional predictive value for employees’ taking charge after controlling for transformational and authentic leadership.

Finally, future research should consider that other mediating mechanisms, such as affective organizational commitment, may play a role in the impact of empowering leadership on taking charge. Some scholars, in studying the relationship between empowering leadership and employees’ three withdrawal behaviors (including lateness, absenteeism, and turnover intention), found that when leaders provide autonomy and developmental support to employees, it positively influences employees’ decisions to stay and participate in the organization, with affective commitment acting as a mediator [77]. The impact of transformational leadership on innovative behavior can be better mediated through affective commitment [84]. Calling-oriented employees influence their taking charge through affective organizational commitment [85]. Research has also shown that ambition is positively correlated with higher affective organizational commitment, rather than achievement pursuit, especially when people perceive more organizational career opportunities [64]. Additionally, some scholars have found that taking charge is positively correlated with employees’ job satisfaction and affective organizational commitment [14]. These findings highlight the importance of affective organizational commitment for both organizational and personal development, providing insights for future research to further explore the effects of empowering leadership and the antecedents of taking charge.

## 6. Conclusions

Mobilizing employees’ initiative and enthusiasm for promoting functional changes within an organization is vital for both the sustainable development of the enterprise and the personal career advancement of employees. This study emphasizes the importance of empowering leadership in enhancing organizational sustainability. Additionally, creating an environment that leverages individual strengths can significantly promote long-term sustainability of the organization. This suggests that organizations should focus on establishing development strategies based on employee strengths under an empowering leadership framework, which is key to achieving sustainable success, especially for employees with high ambitions. Despite existing studies exploring the relationship between empowering leadership and employees’ taking charge behavior, the behavioral mechanisms underlying this relationship remain unclear. This study, for the first time within the framework of COR theory, connects empowering leadership with employees’ taking charge behavior. The findings indicate that empowering leadership facilitates employees’ taking charge behavior, with employees’ use of strengths playing a positive mediating role. Furthermore, we discovered that employees’ work ambition enhances the relationship between empowering leadership, the use of strengths by employees, and their taking charge behavior. This research advances the theory and study of empowering leadership and provides new insights into the drivers of employees’ taking charge behavior. It is recommended that organizations focus on leveraging employees’ strengths and encourage providing resource support to highly ambitious employees. This can more effectively support employees’ career development and enhance their well-being, not only improving long-term job satisfaction and retention rates but also enabling them to play a more active role in the organization’s sustainable development.

## Figures and Tables

**Table 1:** Sample distribution.

Characteristics	Categories	No. of Respondents	(%)
Age	20–29 years	114	34.23
	30–39 years	175	52.55
	40–49 years	39	11.71
	50–59 years	5	1.50
Gender	Male	145	43.54
	Female	188	56.46
Education	Under bachelor’s degree	18	5.41
	Bachelor’s degree	246	73.87
	Master’s degree	64	19.22
	PhD	5	1.50
Tenure	0–5 years	123	36.94
	6–10 years	112	33.63
	11–20 years	82	24.62
	21–30 years	13	3.90
	More than 30 years	3	0.90
Job level	Ordinary employees	175	52.55
	Line leader	76	22.82
	Middle leader	82	24.62

Note: N = 333.

**Table 2:** Results of CFAs: comparison of measurement models.

Models	$\chi^{2}$	df	$\chi^{2}/df$	RMSEA	CFI	TLI	IFI	${∆\chi }^{2}(∆df)$
Four-factor model (baseline model)	786.65	344	2.29	0.06	0.91	0.90	0.91	
Three-factor model ^a^	857.06	347	2.47	0.07	0.89	0.89	0.90	70.41 *** (3)
Two-factor model ^b^	1211.97	349	3.47	0.09	0.82	0.81	0.82	425.32 *** (5)
Single-factor model ^c^	1503.12	350	4.30	0.10	0.76	0.74	0.76	716.47 *** (6)

Note: ^a^ strengths use and taking charge merged; ^b^ empowering leadership and ambition merged, and strengths use and taking charge merged; ^c^ all variables merged. *** *p* < 0.001.

**Table 3:** Means, standard deviations, and correlations.

Variable	M	SD	1	2	3	4	5	6	7	8
1. Gender	0.56	0.5								
2. Age	32.16	6.21	−0.09							
3. Education	1.17	0.53	−0.04	−0.13 *						
4. Tenure	8.46	6.09	−0.08	0.94 **	−0.24 **					
5. Job level	0.72	0.83	−0.08	0.47 **	0.07	0.46 **				
6.Empowering leadership	3.98	0.61	−0.07	0.20 **	−0.08	0.17 **	0.35 **			
7. Ambition	3.92	0.77	−0.18 **	0.01	0.04	−0.02	0.28 **	0.62 **		
8. Strengths use	4.24	0.5	−0.01	0.16 **	−0.11 *	0.11 *	0.25 **	0.62 **	0.54 **	
9. Taking charge	3.78	0.81	−0.02	0.19 **	−0.06	0.14 **	0.33 **	0.67 **	0.60 **	0.68 **

Note: N = 333, * *p* < 0.05; ** *p* < 0.01, two-tailed.

## Data Availability

The data that support the findings of this study are available from L.K. (alicia25@163.com), upon reasonable request.

## Appendix A. Questionnaire Item Scales

**Empowering Leadership 12-item Scale**

1. My manager helps me understand how my objectives and goals relate to that of the company.
2. My manager helps me understand the importance of my work to the overall effectiveness of the company.
3. My manager helps me understand how my job fits into the bigger picture.
4. My manager makes many decisions together with me.
5. My manager often consults me on strategic decisions.
6. My manager solicits my opinion on decisions that may affect me.
7. My manager believes that I can handle demanding tasks.
8. My manager believes in my ability to improve even when I make mistakes.
9. My manager expresses confidence in my ability to perform at a high level.
10. My manager allows me to do my job my way.
11. My manager makes it more efficient for me to do my job by keeping the rules and regulations simple.
12. My manager allows me to make important decisions quickly to satisfy customer needs.

**Ambition 5-item Scale**

1. I am ambitious.
2. I strive for success.
3. I have challenging goals.
4. For me, it is very important to achieve outstanding results in my life.
5. For me, it is very important to accomplish great things.

**Strengths Use 5-item Scale**

1. In my job, I make the most of my strong points.
2. I organize my job to suit my strong points.
3. I capitalize on my strengths at work.
4. I seek opportunities to do my work in a manner that best suits my strong points.
5. In my job, I try to apply my talents as much as possible.

**Taking Charge 6-item Scale**

1. I often try to change how his or her job is executed in order to be more effective.
2. I often try to bring about improved procedures for the work unit or department.
3. I often try to institute new work methods that are more effective for the company.
4. I often try to correct a faulty procedure or practice.
5. I often try to implement solutions to pressing organizational problems.
6. I often try to introduce new structures, technologies, or approaches to improve efficiency.
